# Decreased mortality seen in rifampicin/multidrug-resistant tuberculous meningitis treated with linezolid in Shenzhen, China

**DOI:** 10.1186/s12879-021-06705-4

**Published:** 2021-09-28

**Authors:** Mu-Tong Fang, You-Feng Su, Hui-Ru An, Pei-Ze Zhang, Guo-Fang Deng, Hou-Ming Liu, Zhi Mao, Jian-Feng Zeng, Guobao Li, Qian-Ting Yang, Zhong-Yuan Wang

**Affiliations:** 1 National Clinical Research Center for Infectious Disease (Shenzhen), Guangdong Provincial Clinical Research Center for Infectious Diseases (Tuberculosis), Shenzhen Third People’s Hospital, Southern University of Science and Technology, Shenzhen, 518112 China; 2 Tuberculosis Sector of 8th Medical Center of Chinese PLA General Hospital, Beijing, China

**Keywords:** Tuberculous meningitis, Linezolid, Blood–brain barrier, Rifampicin/multidrug-resistant TBM

## Abstract

**Background:**

The morbidity of rifampicin/multidrug-resistant tuberculous meningitis (RR/MDR-TBM) has shown an increasing trend globally. Its mortality rate is significantly higher than that of non-rifampicin/multidrug-resistant tuberculous meningitis (NRR/MDR-TBM). This article aimed to explore risk factors related to RR/MDR-TBM, and compare therapeutic effects of linezolid (LZD)- and non-linezolid-containing regimen for RR/MDR-TB patients in Shenzhen city. Furthermore, we aimed to find a better therapy for pathogen-negative TBM with RR/MDR-TBM related risk factors.

**Methods:**

We conducted a retrospective study enrolling 137 hospitalized cases with confirmed TBM from June 2014 to March 2020. All patients were divided into RR/MDR-TBM group (12 cases) and NRR/MDR-TBM group (125 cases) based on GeneXpert MTB/RIF and (or) phenotypic drug susceptibility test results using cerebral spinal fluid (CSF). The risk factors related to RR/MDR-TBM were investigated through comparing clinical and examination features between the two groups. The mortality rate of RR/MDR-TBM patients treated with different regimens was analyzed to compare their respective therapeutic effects. A difference of *P* < 0.05 was considered statistically significant.

**Results:**

Most patients (111/137, 81%) were from southern or southwestern China, and a large proportion (72/137, 52.55%) belonged to migrant workers. 12 cases were RR/MDR-TBM (12/137, 8.8%) while 125 cases were NRR/MDR-TBM (125/137, 91.2%). The proportion of patients having prior TB treatment history in the RR/MDR-TBM group was significantly higher than that of the NRR/MDR-TBM group (6/12 vs. 12/125, 50% vs. 10.5%, *P* < 0.01). No significant difference was observed on other clinical and examination features between the two groups. Mortality was significantly lower in RR/MDR-TBM patients on linezolid-containing treatment regimen than those who were not (0/7 versus 3/5, 0% versus 60%, *P* = 0.045).

**Conclusions:**

The main related risk factor of RR/MDR-TBM is the history of anti-tuberculosis treatment. Linezolid-containing regimen appears to lower mortality rate of RR/MDR-TBM significantly in our study. We think Linezolid should be evaluated prospectively in the treatment of RR/MDR-TBM.

## Background

Tuberculous meningitis (TBM) is the most fatal form of tuberculosis, especially in children or adults co-infected with HIV. Even with the use of standardized anti-tuberculosis (TB) treatment, mortality can still reach as high as 20%–50%, and nearly half of the survivors will develop serious central nervous system sequelae [1, 2]. In recent years, an increase in the number of drug-resistant tuberculous meningitis cases had been reported. Compared to non-drug-resistant tuberculous meningitis, drug-resistant tuberculous meningitis, especially rifampicin/multidrug-resistant tuberculous meningitis (RR/MDR-TBM) is more lethal with increased medical cost for the patient [3, 4]. China is the world's second largest country with a high burden of RR/MDR-TB, the estimated number of people infected with RR/MDR-TB in 2019 was about 65,000 (about 14% of the world total). RR/MDR-TB accounted for 7.1% and 23% in newly diagnosed and previously treated tuberculosis patients respectively [5]. As the most lethal form of tuberculosis, the incidence of TBM constituted about 1% of all tuberculosis cases worldwide [6], and about 7.23% of extrapulmonary tuberculosis cases in China [7]. However, the incidence of RR/MDR-TBM and its related risk factors remains unclear. Herein, we conducted a retrospective study in which 137 confirmed TBM patients were enrolled. These patients were hospitalized in the Third People’s Hospital of Shenzhen from June 2014 to March 2020. The hospital is the only designated hospital for the treatment of TBM patients in Shenzhen city. Shenzhen is a megacity with a long-term lived population of more than 13 million, with a large floating population of migrant workers from other cities, especially the southern and southwestern provinces of China. Thus TBM cases being treated in our hospital can reflect the general situation of the disease in south and southwest China to some extent. We compared the related characteristics (including demographic, clinical and examination characteristics) between RR/MDR-TBM and non-rifampicin/multidrug-resistant tuberculous meningitis (NRR/MDR-TBM) patients to explore risk factors associated with RR/MDR-TBM. Furthermore, we compared the effects of linezolid- (LZD) and non-linezolid-containing (non-LZD) regimens on the prognosis of RR/MDR-TBM. On this basis, we discussed the empirical treatment regimen for pathogen-negative TBM with RR/MDR-TBM related risk factors. We hope it will help improve the prognosis of RR/MDR-TBM and pathogen-negative TBM in China.

## Method

**Fig. 1:**
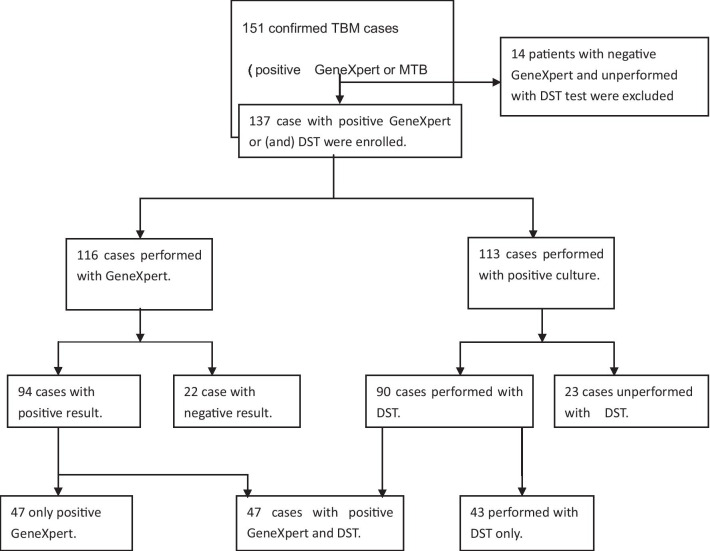
The enrollment of the patients. TBM: tuberculous meningitis. MTB: *Mycobacterium tuberculosis*. DST: drug sensitivity test.Unperformed with DST: DST was not performed when MTB culture was positive (as the patient had died or abandoned treatment and discharged before the lab informed a positive culture)

Patients’ enrollment and data collection:

1. Enrollment process: This study adopted a retrospective method. We collected the data of 151 tuberculous meningitis patients who were hospitalized and met the diagnostic criteria for confirmed tuberculous meningitis from June 2014 to March 2020. Among them, 14 patients who did not perform GeneXpert MTB/RIF and drug susceptibility test (DST) were excluded. Finally, 137 patients were enrolled in the study (Fig. 1), including 98 males and 39 females, age ranged from 2 to 76 years, with a median age of 29 years. We collected the demographic/clinical and examination characteristics of these patients from the medical record through the hospital information system (HIS) and the hospital laboratory information system (LIS), and confirmed the survival status of discharged patients through phone and medical follow-up.
2. Entry criteria: Met the diagnostic criteria for definite tuberculous meningitis [8], that is, in addition to the clinical manifestations of TBM and abnormality of cerebrospinal fluid (CSF), the patient’s CSF examination met at least one of the following two criteria: 1. GeneXpert MTB/RIF (GeneXpert) positive nucleic acid test for *Mycobacterium tuberculosis* (MTB). 2. BACTEC MGIT 960 positive culture identifying as MTB, with a phenotypic drug sensitivity test (DST) performed simultaneously. Only one pathogen-positive CSF result was analyzed for each patient.
3. Diagnostic criteria for RR/MDR TBM cases: Met the diagnostic criteria for definite tuberculous meningitis case and fulfilled at least one of the following two criteria: 1. Positive GeneXpert test indicating resistance to rifampicin (RIF) 2. CSF MTB positive culture, and DST showing resistance to rifampicin and resistance or sensitivity to isoniazid.
4. We described the demographic features of TBM patients according to the results of GeneXpert and/or DST in CSF. All patients were divided into RR/MDR group and NRR/MDR group, then we compared the differences of the main clinical and examination characteristics between the two groups. On this basis, we explored the associated risk factors of RR/MDR TBM. Finally, we compared the differences in mortality between the two groups and the impact of different regimen on the prognosis of RR/MDR-TBM.

### Laboratory examination

1. Method of obtaining CSF specimen: Lumbar puncture was performed within 24 h from admission. For patients whose CSF was acquired through lateral ventricle drainage, the pressure of CSF was measured. The CSF specimen was submitted at once after collection for tests such as white blood cell count and classification, biochemistry, acid-fast bacillus (AFB), GeneXpert, MTB culture and species identification, Cryptococcus membrane polysaccharide antigen, bacteria and fungi smears and culture. The volume of CSF used for GeneXpert and MTB culture was 1–2 ml.
2. Laboratory examination method:
  1. GeneXpert MTB/RIF: According to literature and instrument operating instructions [9, 10].
  2. BACTEC MGIT 960 Mycobacterium Culture Identification System for cerebrospinal fluid MTB: according to literature [11].
  3. The phenotypic drug susceptibility test (DST) of MTB: absolute concentration method was adopted [12]. The test covered a minimum of 4 drugs (isoniazid, rifampin, ethambutol, streptomycin), including low and high concentration.
  4. Acid-fast staining, white blood cell count and classification, biochemistry of CSF and blood routine, blood biochemistry, blood tuberculosis interferon release assay (IGRA) and HIV antibody were operated in accordance with the instruction of the instrument and kit.
3. Anti-tuberculosis treatment regimen: Non-RR/MDR-TBM group: Anti-tuberculosis treatment regimen included: Isoniazid (10–15 mg/Kg/day, the maximum dose was 0.9 g/day, administered intravenously during hospitalization, then changed to oral administration after discharge); Rifampicin (10-15 mg/Kg/day, maximum 0.6 g/day, administered intravenously during hospitalization, and changed to oral administration after discharge); Pyrazinamide (25–30 mg/Kg/day, 1.5–2.0 g for adults, oral or nasal administration); ethambutal (15 mg/kg/day, 0.75–1.0 g for adults, oral or nasal administration). Some patients were supplemented with streptomycin injection or levofloxacin (or moxifloxacin) on top of the above. Full course of treatment for NRR/MDR-TBM is 12–18 months except in the patient’s death. RR/MDR-TBM group: 7 patients were treated with LZD-containing regimen (LZD 1200 mg/day, intravenously or orally in the first 2 weeks of hospitalization, then changed to oral administration 600 mg/day, for a course of 3 to 18 months). The regimen also included levofloxacin (or moxifloxacin) and 2–4 of the following drugs: cycloserine, pyrazinamide, high-dose isoniazid, amikacin, protionamide, in dosage in accordance with the nation’s management guideline for RR/MDR-TB). Five patients received the non-LZD-containing regimen in the early stage which included the following 4–6 drugs: levofloxacin (moxifloxacin), pyrazinamide, high-dose isoniazid, rifampicin, amikacin, protionamide, cycloserine and ethambutol. The scheduled course of treatment for RR/MDR-TBM was a minimum of 24 months except for the patient’s death. In addition to anti-tuberculosis therapy, all patients were treated with glucocorticoids for up to 3 months and other adjuvant treatments deemed necessary such as mannitol for lowering intracranial pressure. Several were performed with lateral ventricle drainage.
4. Treatment outcome: expressed as survival status at the end of the treatment course [13]. In the case the course of treatment had not been finished, the survival status at 6 months of treatment was taken as treatment outcome. Treatment outcome was expressed as death or survival.
5. Statistical methods: qualitative data were expressed as percentages; quantitative data were expressed as mean or median ± standard deviation. Rates were compared using chi-square test or Fisher's exact probability method, and quantitative data were compared using t-test or rank-sum test. The difference was considered statistically significant when P < 0.05. The statistical software used is Prism 5.0 software package.

## Results

1. Birthplace and occupational distribution of the TBM patients: most of the patients came from southern and southwestern provinces of China, such as Guangdong province (18.3%) and Guangxi Zhuang Autonomous Region (9.5%). Migrant workers (72 cases, 52.55%) were the most common occupation for these patients (Table 1).
2. Drug resistance profile of the TBM patients: the difference of drug resistance between new cases and previously treated cases was (6/119, 5.0%) and (6/18, 33.3%) for rifampicin and (3/119, 2.5%) and (4/18, 22.2%) for multidrug respectively. Both were shown to be significant statistically (P < 0.01) (Table 2).
3. Comparison of clinical features and CSF examination features between RR/MDR-TBM group and NRR/MDR-TBM groups: 50% of the patients in the RR/MDR-TBM group had a history of anti-tuberculosis treatment (6/12), while only 10.5% were reported in the NRR/MDR-TBM group (6/125). The difference between the two groups was statistically significant (P < 0.01). Difference in other factors showed no statistical significance (Table 3).
4. Comparison of mortality between RR/MDR-TBM group and NRR/MDR-TBM group: the difference of mortality between RR/MDR-TBM group and NRR/MDR-TBM group showed no statistical significance, regardless of treatment completion and follow-up status. See Table 4.
5. Comparison of mortality between RR/MDR-TBM patients groups treated with different regimens: among RR/MDR-TBM patients, the mortality of patients treated with LZD-containing regimen at early treatment stage was significantly lower compared with those without (P = 0.045), see Table 5–6.

**Table 1:** The native place and occupational distribution of the TBM patients

	Cases number	Ratio
Native place		
Shenzhen city	10	7.3%
Guangdong province (except Shenzhen)	25	18.3%
Guangxi Zhuang Autonomous Region	13	9.5%
Hunan province	12	8.8%
Jiangxi province	11	8.0%
Hubei province	8	5.8%
Sichuan province	13	9.5%
Guizhou province	10	7.3%
Chongqing city	9	6.6%
Unknown place	7	5.1%
Other 11 province or city	19	13.9%
Occupation		
Migrant worker	72	52.6%
Staff (white collar)	15	11.0%
Unemployment or housework	12	8.8%
Retirement	10	7.3%
Self-employed people	8	5.8%
Homeless people	8	5.8%
Students	6	4.4%
Imprisoned	3	2.2%
Farmer	3	2.2%

**Table 2:** The drug resistant profile of the TBM patients

D-R type	Total (n = 137)	New cases (n = 119)	Previously treated cases (n = 18)	*P* value
INH-R	3/90 (3.3%)	2/79 (2.5%)	1/11 (9.1%)	0.330
RIF-R	12/137 (8.8%)	6/119 (5.0%)	6/18 (33.3%)	< 0.01
MDR	7/137 (5.1%)	3/119 (2.5%)	4/18 (22.2%)	< 0.01

D-R type, drug resistant type; INH-R, isonaizid resistant; RIF-R, rifampicin resistant; MDR, multidrug resistant; INH resistant plus RIP resistant; New cases, a newly registered episode of TB in a patient who has never been treated for TB or has taken anti-TB medicines for less than 1 month. Previously treated cases: patients who have received 1 month or more of anti-TB medicines in the past

**Table 3:** The comparison of the features between RR/MDR-TBM and NRR/MDR-TBM groups

	TBM	RR/MDR-TBM	NRR/MDR-TBM	P value
Total	137	12	125	
Male	98	11	87	0.178
Age(year)	29	29 ± 18	29 ± 12.25	0.927
TB history	18	6	12	< 0.01
Miliary PTB	71	6	65	0.895
Active PTB	55	6	49	0.466
Extrapulmonary TB	44	4	40	0.819
HIV co-infection	17	2	15	0.992
Type-II diabetes	7	1	6	0.595
Course of disease (days from onset to admission)	10 ± 10	3 ± 12	10 ± 10	0.314
Headache	85	5	80	0.128
Fever	93	8	85	0.433
Vomiting	40	2	38	0.505
Convulsion	12	3	9	0.123
Consciousness disorder	56	7	49	0.198
Neck stiff	114	10	105	0.725
Cranial nerve impairment	23	2	21	0.695
Pathological sign	33	6	33	0.084
Paralysis	9	1	8	0.725
GCS score	13.13 ± 2.57	12.25 ± 2.83	13.22 ± 2.54	0.215
BMRC gradeI	76	4	72	0.190
BMRC gradeII	33	4	29	0.667
BMRC grade III	28	4	24	0.432
Death/total	24/87	3/12	21/75	0.895
Hyponatremia	35	5	30	0.180
IGRA (positive/total)	98/128	8/11	90/117	0.954
CD4 T cell count	221 ± 189	213 ± 188	280 ± 196	0.156
CSF examination				
Elevated CSF pressure	72	7	65	0.675
WBC(10^6^/L)	231 ± 87.5	104 ± 442.3	287 ± 389	0.060
NEUT%	56.8 + 24.8	57.5 ± 24.9	56.8 ± 24.9	0.961
PRO (mg/L)	1843 ± 1201	1885 ± 1222	1407 ± 71.4	0.167
CL (mmol/L)	110 ± 12.3	110.6 ± 12.5	112.7 ± 10.96	0.385
GLU (mmol/L)	1.9 ± 1.2	1.9 ± 1.2	2.0 ± 1.0	0.522
ADA (U/L)	8.7 ± 15.6	5.4 ± 4.2	9.0 ± 16.3	0.172

(N)RR/MDR TBM, (non) rifampicin/multidrug-resistant tuberculous meningitis

PTB, pulmonary tuberculosis; HIV, human immunodeficiency virus; GCS, Glasgow Coma Scale; BMRC, the modified British Medical Research Council; Grade I (GCS 15; no focal neurological signs), grade II (GCS 11–14, or 15 with focal neurological signs), and grade III (GCS ≤ 10) disease. IGRA, interferon gamma release assay; CSF, cerebrospinal fluid; WBC, white blood cell; NEUT, neutrophilic granulocyte; PRO, total protein; CL, chloride; GLU, glucose; ADA, adenosine deaminase

**Table 4:** The comparison of the mortality between RR/MDR-TBM and NRR/MDR-TBM groups

	Total	RR/MDR-TMB	nRR/MDR-TBM	P value
Deaths	24	3	21	
Survivors	63	9	54	
Loss to follow-up	50	0	50	
Mortality1	27.6%	25.0%	28.0%	0.895
Mortality2	17.5%	25.0%	16.8%	0.752
Mortality3	54.0%	25.0%	56.8%	0.071

Mortality 1: excluding those lost to follow-up. Mortality 2: including those lost to follow-up and assuming all of them survived. Mortality 3: including those lost to follow-up and assuming all of them demised

**Table 5:** The mortality of the RR/MDR-TBM patients treated with different regimens

	Linezolid containing regimen	Non-linezolid regimen	P value
Total cases	7	5	
Death	0	3	
Mortality	0%	60%	0.045

**Table 6:** The length of LZD and outcomes for RR/MDR-TBM patients

Group	Case	Age range (y)	Length of disease(d)	BMRC	HIV	Mi-TB	Regimen	Length of lzd(m)	Outcomes
LZD	Case1	15–24	3	III	−	+	LZD-INH-PZA-Mfx-Cs	3	Survival
	Case2	25–34	7	II	−	−	LZD-Mfx-CS-PZA-INH-Pto	6	Survival
	Case3	25–34	14	III	−	−	LZD-Mfx-Pto-PZA-Am	6	Survival
	Case4	25–34	1	II	−	+	LZD-INH-PAZ-EMB-Lfx-Am	6	Survival
	Case5	25–34	3	I	+	+	LZD-Mfx-Pto-PZA-Am	8	Survival
	Case6	15–24	1	I	+	+	LZD-Mfx-PZA-Pto-Am	9	Survival
	Case7	35–44	15	I	−	−	LZD-INH-PZA-Mfx-CS	10	Survival
Non-LZD	Case8	24–34	77	II	−	−	INH-RIF-PZA-EMB-Lfx		Death
	Case9	15–24	3	II	−	−	INH-RIF-PZA-EMB		Survival
	Case10	25–34	21	III	−	+	INH-PZA-Mfx-Pto-Am		Survival
	Case11	45–54	1	I	−	+	INH-RIF-PAZ-EMB		Death
	Case12	45–54	3	III	−	−	INH-PZA-EMB-Lfx-Am		death

F, female; m, male; y, year; d, day; m, month; HIV, human immunodeficiency virus; mi-TB, military tuberculosis; BMRC, the modified British Medical Research Council. Grade I (GCS 15; no focal neurological signs), grade II (GCS 11–14, or 15 with focal neurological signs), and grade III (GCS ≤ 10) disease. INH, isoniazid; RIF, rifampicin; PZA, pyrazinamide; EMB, ethambutol; Lfx, levofloxacin; Mfx, moxifloxacin; Am, amikacin; Cs, cycloserine; LZD, linezolid; Pto, prothionamide

## Discussion

The study showed that most of the TBM patients being treated in our hospital were from the south and southwest of China, with a large proportion of them being migrant workers. RR/MDR-TBM accounted for a relatively high proportion among them. The history of anti-tuberculosis treatment was found to be one of the related risk factors for RR/MDR-TBM. Treatment regimen comprising linezolid seemed to be decreasing the mortality of RR/MDR-TBM significantly.

The incidence of TB in the south and southwest of China has been reported to be higher than other regions of the country. This was found to be consistent with the findings in our study based on patient’s demographic data. More than half of the patients in our study were migrant workers. The high incidence of TBM among them may be attributed to poor nutrition and deprivation of rest resulted from a low income and long working hours. A weak or impaired immune system fails to provide proper defense against *Mycobacteria tuberculosis.*

More and more RR/MDR-TBM had been reported in recent years. Some small sample size studies had shown that RR/MDR-TBM accounted for 12%–39.29% of TBM patients in western China, and the ratio of isoniazid-resistant TBM even reached as high as 64.29% [14, 15]. Even in children with first-time TB infection, MDR and XDR accounted for 7.2% [16]. Although the sample size of these studies was small, it still indicated that RR/MDR-TBM is not a rare disease in China. Reports from other countries also suggested that RR/MDR-TBM constituted a relatively high proportion of TBM patients [3, 4, 17, 18]. Our study showed the proportion of RR/MDR-TMB patients in Shenzhen is also high (12/137, 8.8%). In terms of proportion, RR/MDR-TMB in previously treated cases is significantly higher than that of primary infection cases (33.3% vs. 5.0%, P < 0.01), which is in line with the situation of RR/MDR-PTB. This can be explained by the fact that TBM is developed from PTB. Therefore, the history of anti-tuberculosis treatment should be considered as the main risk factor for RR/MDR-TBM.

A series of literature suggested that the risk factors associated with RR/MDR pulmonary tuberculosis included previous history of anti-tuberculosis treatment, HIV infection, diabetes and other factors [4, 5, 17–19]. In our study, only the history of anti-TB treatment showed a significant statistical difference between the RR/MDR-TBM and NRR/MDR-TBM groups (6/12 vs. 18/119, P < 0.01), while the difference of other factors was statistically insignificant. The reason maybe partly due to the low ratio of comorbidity such as HIV and diabetes in our study. Therefore, TBM patients with prior anti-TB treatment history are more susceptible to be RR/MDR-TBM. Treatment outcomes of RR/MDR-TBM can be extremely unfavorable. For example, it was reported the mortality of RR/MDR-TBM treated with first-line anti-tuberculosis drugs could reached as high as 100% [4]. Therefore, the early diagnosis of tuberculous meningitis and the evaluation of its drug resistance appeared critical for formulating an effective regimen. In our study, the NRR/MDR-TBM patients were treated with high dose of INH and standard dose of RIF (10 mg/kg, the maximum dose is 0.6 g), PZA, EMB according to the national tuberculosis management guideline. Some of these patients’ regimen were supplemented with another drug such as levofloxacin or moxifloxacin. Some research showed that high dose of RIF (20–35 mg/kg administered orally, and standard dose of INH) was able to improve treatment outcomes due to a higher RIF concentration in plasma and CSF, especially when RIF was administered intravenously [20, 21]. Other research did not reach the same conclusion above, though [18]. We believe that high dose of INH is more important than high dose of RIF for the treatment of TBM. First because INH has excellent early bactericidal activity (EBA), and the genotype of N-acetyltransferase type 2 (NAT2) for the majority of Chinese are rapid acetylators. Recently, a study conducted by Jing had shown that about 36.0%, 42.7% and 21.3% Chinese population are fast, intermediate and slow acetylators respectively according to NAT2 genotype. A population pharmacokinetic (PPK) model for isoniazid among Chinese tuberculosis patients was established for the first time which suggested an approximated dosage of 800 mg/day, 500 mg/day or 300 mg/day for fast, intermediate and slow acetylators, respectively, in order to achieve effective and safe plasma concentration [22]. Standard dose of INH for most Chinese TBM patients will only result in low drug concentration in plasma and CSF, regardless of its potent permeability through the blood brain barrier (BBB). However, a high dose of INH plus RIF will increase the incidence of hepatotoxicity easily. Thus, we chose high dose of INH and standard dose of RIF, of which RIF was administered intravenously instead of orally to increase drug level in plasma and CSF. According to the previous report, 13 mg/kg of IV RIF can achieve an equivalent of AUC0-24 h which drives rifampicin effect and a higher Cmax compared to 20 mg/kg of orally administered RIF [20].

Many patients in the NRR/MDR-TBM group were lost to follow-up. Reasons behind may include: an invalid contact number due to an expired calling card or the patient leaving the city to return to his/her birthplace due to loss of job. Secondly, we cannot rule out that possibility of the telephone number being cancelled was due to the patients’ death. So we assume two extreme situations, that is, patients who were lost to follow-up in this study either survived or died (the real situation should locate between these two extremes). And the results showed that in any case, the difference of mortality between the RR/MDR-TBM group and NRR/MDR-TBM group remained insignificant, indicating that after timely and proper anti-TB treatment, the mortality of RR/MDR-TBM patients was not higher than that of NRR/MDR-TBM.

Among RR/MDR-TBM patients, mortality rate in patients on treatment regimen with LZD was significantly lower than those without (see Tables 5–6). This showed that LZD may improve treatment outcome due to its strong early bactericidal activity (EBA) and excellent permeability through the blood–brain barrier (BBB). Its CSF-to-serum ratio of the areas under the curves nearly reached 1.0 [23]. Some retrospective studies had indicated that LZD was able to manifest satisfactory effect to life-threatening TBM and improved their early outcomes for both children and adult TBM patients. In Li’s research involving 86 children with TBM, 32 (88.9%) in 36 LZD-treated hospitalized cases and 35 (70%) of 50 control group had favorable outcomes (p = 0.037). In addition, there was no significant difference in the frequency of adverse effect between the 2 groups [24]. In another research conducted by Sun et al. 16 LZD-treated TBM patients (BMRC grade II or III) achieved a faster and higher percentage of Glasgow coma scale and temperature recovery, a higher CSF/blood glucose ratio, and lower CSF white blood cell counts than the control group did (p < 0.05) [25]. But in these two studies, DST or fast detection of MTB nucleic acid such as Genexpert MTB/RIF had not been performed, so the proportion of RR/MDR-TBM was unclear. The addition of a single drug to a failing regimen is perilous, as it may induce further resistance. Meanwhile, the case number in Sun et al.’s study is too small to reach a good representation of evidence-based conclusion and the study only evaluated interim outcome, long term outcome was not known. Only some case reports observed favorable long term outcomes of RR/MDR-TBM patients who received LZD treatment [26, 27]. To our knowledge, our cohort study appears to be the first one to evaluate the long term outcomes of LZD-containing regimen on RR/MDR-TBM patients.

For pathogen-positive TBM cases, anti-tuberculosis treatment regimen can be formulated based on the results of drug sensitivity test. Unfortunately, only less than 40% of total non-HIV TBM cases in this study were tested pathogen-positive even with the use of assays with high sensitivity and specificity like GeneXpert RIF/MTB or GeneXpert Ultra [28]. For pathogen-negative cases, it is advisable to initiate empirical anti-tuberculosis treatment as early as possible. The regimen consists of first-line drugs (isoniazid, rifampicin, pyrazinamide, ethambutol), which, however, has yielded unsatisfactory outcome on drug-resistant TBM, especially RR/MDR-TBM. Even when fluoroquinolones is added to the regimen and the dose of rifampicin is increased, it can only lower the mortality of isoniazid-resistant TBM but not that of RR/MDR-TBM [3, 18]. Previous literatures and our study showed that the proportion of RR/MDR-TBM in pathogen-positive TBM patients with prior TB treatment history is very high in China. We believe the same also exists in pathogen-negative TBM patients with previous TB treatment history. Treatment regimen composes of first-line anti-tuberculosis drugs alone will obviously worsen their outcome. So for pathogen-negative TBM cases treated previously, linezolid may be beneficial when it is added to an empirical treatment regimen. But this should be evaluated by large scale and prospective study. Meanwhile the regimen should contain other effective second-line drugs, such as levofloxacin (moxifloxacin) or cycloserine [29]. The challenge, however, is whether RIF should be included in the regimen, since more than 60% of the previously treated TBM patients in our study showed sensitivity to RIF, and a large proportion of them complicated with PTB, so RIF and other first-line drugs should also be included.

There are some limitations in this study. First of all, it is unclear whether the drug resistance profile of pathogen-negative TBM patients is exactly the same as that of pathogen-positive TBM patient. Meanwhile, DST did not test sensitivity to some second line drugs such as linezolid, fluroquinolones and cycloserine. Secondly, the number of RR/MDR-TBM cases was very small, so the lower mortality was not necessarily due to LZD but maybe by chance. It is necessary to further increase case number for a prospective multicenter cohort study to evaluate the efficacy and adverse effects of LZD-containing regimens. Finally, the high proportion of lost to follow-up patients in the NRR/MDR-TBM group also made it difficult to analyze the results, although we had made appropriate assumptions.

## Conclusions

The history of anti-tuberculosis treatment is considered to be the main related risk factor of RR/MDR-TBM. Notwithstanding our small sample size retrospective study, LZD-containing regimen seemed to lower the mortality of RR/MDR-TBM significantly. We think LZD should be evaluated prospectively in the treatment of RR/MDR TBM.

## Data Availability

Some of them have been provided in the manuscript, the rest was available from the corresponding author on reasonable request.

## Abbreviations

ADA: Adenosine deaminase
AFB: Acid fast bacilli
BBB: Blood brain barrier
BMRC: The modified British Medical Research Council
CL: Chloride
CSF: Cerebrospinal fluid
DST: Drug susceptibility test
EBA: Early bactericidal activity
GLU: Glucose
GSC: Glasgow Coma Scale
HIS: Hospital information system
HIV: Human Immunodeficiency Virus
INH: Isoniazid
LIS: Laboratory information system
LZD: Linezolid
MTB: *Mycobacterium tuberculosis*
NRR/MDR-TBM: Non-rifampicin/multidrug-resistant tuberculous meningitis
PRO: Protein
PTB: Pulmonary tuberculosis
RIF: Rifampicin
RR/MDR-TBM: Rifampicin/multidrug-resistant tuberculous meningitis
TB: Tuberculosis
WHO: World Health Organization
